# A Prospective Cohort Study Assessing the Impact of Fixed Orthodontic Appliances on Saliva Properties and Oral Microbial Flora

**DOI:** 10.3290/j.ohpd.b898961

**Published:** 2021-01-26

**Authors:** Georgios Kouvelis, Aikaterini Papadimitriou, Kyriakoula Merakou, Ioannis Doulis, Stergios Karapsias, Dimitrios Kloukos

**Affiliations:** a Researcher, Department of Orthodontics and Dentofacial Orthopedics, 251 Hellenic Airforce V.A. General Hospital, Athens, Greece. Performed all clinical assessments and measurements, wrote the manuscript, prepared all figures, reviewed the manuscript.; b Researcher, Department of Orthodontics and Dentofacial Orthopedics, 251 Hellenic Airforce V.A. General Hospital, Athens, Greece. Performed all clinical assessments and measurements, wrote the manuscript, prepared all figures, reviewed the manuscript.; c Professor, Department of Public and Administrative Health, National School Of Public Health, Athens, Greece. Oversaw the project and assisted with writing the the manuscript, reviewed the manuscript.; d Researcher, Department of Orthodontics and Dentofacial Orthopedics, 251 Hellenic Airforce V.A. General Hospital, Athens, Greece. Assisted with interpretation of statistics, reviewed the manuscript.; e Researcher, Department of Microbiology-Biopathology, 251 Hellenic Airforce V.A. General Hospital, Athens, Greece. Performed all microbiological assessments, reviewed the manuscript.; f Researcher, Department of Orthodontics and Dentofacial Orthopedics, 251 Hellenic Airforce V.A. General Hospital, Athens, Greece; Department of Orthodontics and Dentofacial Orthopedics, School of Dental Medicine, University of Bern, Bern, Switzerland. Performed all clinical assessments and measurements, wrote the manuscript, prepared all figures, oversaw the project, assisted with writing the manuscript, reviewed the manuscript.

**Keywords:** microbial flora, orthodontics, saliva

## Abstract

**Purpose:**

Orthodontic treatment may introduce a risk to the integrity of enamel due to plaque accumulation and colonisation by oral microbes. This prospective cohort study observed the effect of fixed, self-ligating orthodontic appliances on saliva properties and oral microbial flora.

**Materials and Methods:**

Thirty adolescent patients were recruited (13 female, 17 male, mean age 13.97 ± 2.07 years). Saliva samples were collected before placement of fixed orthodontic appliances (T0), and 4 (T1) and 12 (T2) weeks later. Salivary pH, flow rate and buffering capacity were recorded. All saliva samples were cultured on agar plates for 2 days. Salivary prevalence of *Neisseria* spp., streptococci, *Staphylococcus aureus*, coagulase-negative staphylococci and *Candida albicans* were assessed.

**Results:**

High buffering capacity was reported in 21 patients at T0, 22 patients at T1 and in 28 patients at T2. Saliva flow rate also increased over time (7.08 ml/5 min at T0, 7.93 ml/5 min at T1 and 8.35 ml/5min at T2). Mean pH was 7.63 at T0, 7.67 at T1 and 7.78 at T2. There was no evidence that either pH or the number of colonies of any of the microbial species changed over time.

**Conclusion:**

The increased buffering capacity of saliva as well as the salivary flow rate after initial bonding might be protective against the development of dental caries. Current microbial findings indicate that initiation of orthodontic treatment may not be associated with significant changes in oral microbial flora.

Fixed orthodontic appliances may be considered hazardous for enamel integrity due to plaque accumulation and colonisation by oral microbes.^58^ With the placement of such appliances, new retentive areas in the oral cavity become available.^43^ Enamel demineralisation has been reported after bracket insertion,^41^ as soon as one month post-placement.^25^ Fixed orthodontic appliances may, moreover, impede oral hygiene procedures and cause alterations in the oral microflora by reducing pH, as well as by increasing plaque accumulation and the affinity of bacteria to metallic surfaces due to electrostatic reactions.^3^

Increased levels of *S. mutans* and *Lactobacillus* species have been reported to exist in the oral cavity after bonding orthodontic appliances, with a positive correlation between caries and the degree of bacterial infection.^39^ *S. mutans,S. sobrinus* and *Staphylococcus aureus* have also been identified as main contributors to the pathogenesis of caries, and their presence contributes to the risk of enamel demineralisation.^7,29^ Moreover, orthodontic treatment has been associated with an increase in periodontopathic microbes such as *S. mutans, S. viridans* and *Lactobacillus* spp.^6,20,23, 33,40,46,49,57^ However, further research is still required, as new microbes have been associated with caries, such as *Streptococcus salivarius, Streptococcus sobrinus,* and *Streptococcus parasanguinis*.^26^

Saliva possesses the ability to influence oral hygiene. Alterations of buffering capacity, pH and flow rate of saliva play a significant role in proper oral function and the occurrence of caries,^17,32,35^ while orthodontic treatment alters its physical and chemical properties.^19^ As far as salivary properties are concerned, both long- and short-term studies have demonstrated contradictory outcomes about alterations on buffering capacity, pH and flow rate.^5,9,12,14,15,34,36,38,48,53^

The aim of this prospective cohort study was to investigate whether or not fixed orthodontic appliances affect salivary properties, as well as whether they affect certain species of oral microbial flora, such as Neisseriae, *Streptococcus mitis/oralis, Streptococcus mutans,* coagulase-negative staphylococci, *Staphylococcus aureus* and *Candida albicans*. Microbes whose correlation with fixed orthodontic treatment has not yet been examined in detail, such as *Streptococcus sanguinis, S. paransanguinis, S. salivarius* and *S. anginosus*, were also examined.

## Materials and Methods

### Sample Size Calculation

Sample size was calculated using the ‘guided study design’ mode of GLIMMPSE (http://glimmpse.samplesizeshop.org/) for repeated-measures studies. Statistical power was set to 0.8, repeated measures were set to 0, 30 and 90 days. The primary hypothesis was set to treatment-by-time interaction, the statistical test employed was Hotelling-Lawley Trace test, and Type I error was set to 0.05. Finally, correlations were set to LEAR (Linear exponent first-order autoregressive). With these assumptions, a sample size of 30 participants in total was acquired.

### Study Sample

Thirty consecutive orthodontic patients were included in the study (13 females, 17 males, mean age 13.97±2.07 years). The sample for this study was recruited from patients presenting for treatment at the Orthodontic Department of the 251 Air Force General Hospital, Athens, Greece between 2/3/2017 and 30/10/2017. To qualify for enrollment in the study, patients had to meet the following eligibility criteria: adolescents (12-18 years old) of any sex, no reported oral habits affecting periodontal health, including smoking, systemic diseases, or any medication affecting the oral cavity taken within the last 3 months, no teeth with active caries and/or missing teeth due to caries, absence of previous or active periodontal disease, orthodontic treatment plan without tooth extractions and no bands on molars or other auxiliaries.

Approval was obtained from the Hospital Ethics Board before study commencement (251 Hellenic Air Force Hospital/ Scientific Committee/Nr.076/6271/1404/30.3.2017, Athens, Greece), and written informed consent was obtained from all participants included in the study, or their guardians. All research was performed in accordance with relevant guidelines and regulations.

### Clinical Procedures

The patients were assigned to receive fixed orthodontic treatment with self-ligating brackets and Nickel-Titanium (NiTi) archwires in both arches (metallic labial brackets/tubes, Innovation-R and Sentalloy Wire 0.014-inch, both from GAC International; Central Islip, NY, USA) for three months. Each patient received professional oral care and standardised hygiene instructions 3 weeks before the beginning of orthodontic treatment, using a typodont model, with specific attention to fixed appliance care. The bonding procedure was performed with the direct technique using Transbond-XT (3M Oral Care; St Paul, MN, USA). All patients were asked to refrain from eating, drinking, and brushing 2 h prior to all clinical examinations and saliva collection. These procedures were performed in a dental chair between 9:00 and 12:00 a.m. After the completion of orthodontic bonding, all patients again received the same oral hygiene instructions. All experiments were done immediately after saliva collection.

### Flow Rate of Saliva

In order to estimate saliva flow rate, patients were asked to chew a paraffin pellet for 5 min. The patient was instructed not to swallow any of the saliva and collection was made by using sterile urine boxes. After 5 min of stimulated salivation, saliva foams were removed and the procedure was completed by measuring the remaining saliva with single-use syringes.

### Saliva Buffering Capacity

CRT buffer strips were used to measure buffering capacity (Ivoclar Vivadent; Schaan, Liechtenstein). A small quantity of saliva (1 ml) was taken from the urine box with a pipette and placed on the strip. After exactly 5 min, the colour alteration of the strip was observed and recorded. The colours yellow, green and blue indicated low, medium and high buffering capacity, respectively.

### pH Levels

The pH levels of all saliva samples were estimated by using pH-indicator strips (Neutralist, Merck; Lebanon, NJ, USA). According to this method, pH values were determined to an accuracy of 0.5 and a range of 5.0 to 10.0 on the pH scale.

### Microbial Analysis

Microbiological detection of specific saliva flora (Neisseriae, *Streptococcus mitis/oralis, Streptococcus anginosus, Streptococcus mutans, Streptococcus salivarius, Streptococcus sanguinis, Streptococcus parasanguinis,* coagulase-negative staphylococci [CoNS], *Staphylococcus aureus, Candida albicans*) was performed with the conventional microbiological method of quantitative culture. The salivary microbes mentioned above are usually estimated in quantities greater than 100,000 (10^5^) colony forming units (cfu) per ml.^30,52,56^ Thus, all saliva samples underwent a 1000-fold dilution using sterile normal saline (0.9% NaCl). Then, saliva culturing was performed by inoculating 10 μl (0.01 ml) of diluted saliva onto Chapman and chocolate agar plates for two days of incubation at 35ºC. Following this method, each colony appearing on agar plates stood for 1 x 10^5^ cfu per ml, because every colony originated from 0.01 ml saliva already diluted 1000 times (1x1000 / 0.01ml = 1 x 10^5^/ ml). Microbes that grew on Chapman agar plates were recognised as staphylococci. Chocolate agar plates were used to culture streptococci and *Neisseria* species. All microbial colonies were Gram stained. Species identification was performed by current standard methods for staphylococci and streptococci (MicroScan WalkAway, Siemens; Newark, NJ, USA) as well as for Neisseriae (Neisseria 4H, BIORAD; Marnes-la-Coquette, France). Staphylococcal species were finally reported as either *Staphylococcus aureus* or CoNS. Streptococci were reported with their species names, whereas all *Neisseria* species were reported as parts of the Neisseriae family.

### Statistical Analysis

Descriptive statistics were applied and normality assumption was tested with the Shapiro-Wilk test for each timepoint separately. The repeated measurements were tested with the non-parametric Friedman test. Only variables with at least 10 observations with positive findings at any given timepoint were included in the analysis. Statistical significance was corrected via Sidak’s correction α_Sidak_ = 1 – (1 – α)1/m, where α is the statistical significance and m the number of hypotheses tested. Finally, post-hoc analyses were conducted between T0-T1, T0-T2 and T1-T2 measurements using the Wilcoxon signed-rank test. The statistical significance was set at α = 0.05. All statistical analyses were performed and graphical plots constructed using Stata 13.0/SE software (Stata; College Station, TX, USA).

## Results

### Description of the Outcomes

Descriptive statistics for the measurements at T0, T1, and T2 are reported Tables 1, 2, and 3, respectively. All the variables in Table 1 were tested for normality with the Shapiro-Wilk test for any given timepoint. Normality assumption was violated for all tested variables. Therefore, all the respective repeated measurements were analysed with the non-parametric Friedman test. Table 4 displays the buffering capacity measurements for each timepoint. The results of the Friedman test are reported in Table 5. For m=6 (six hypotheses tested) and α = 0.05, statistical significance was corrected to α_Sidak_≈0.009. The results of the Wilcoxon signed-rank tests are given in Table 6.

**Table 1:** Descriptive statistics for T0 phase

Variable	Positive counts	Mean (SD)	min, max	Q1	Median	Q3
Flow rate (ml/5 min)	–	7.08 (4.33)	1, 18	3.8	5.75	10
pH	–	7.63 (0.51)	6, 8	7.5	8	8
*Neisseria* spp. (CFU/ml)	30	2283333 (2785193)	100000, 10000000	500000	1000000	3000000
*S. mitis/oralis* (CFU/ml)	19	3106667 (4677896)	0, 15000000	0	800000	4000000
*S. anginosus* (CFU/ml)	6	1083333 (2532830)	0, 10000000	0	0	0
*S. mutans* (CFU/ml)	1	333333.3 (1825742)	0, 10000000	0	0	0
*S. salivarius* (CFU/ml)	6	490000 (1126285)	0, 4000000	0	0	0
*S. sanguinis* (CFU/ml)	1	666666.7 (3651484)	0, 20000000	0	0	0
S. parasanguinis (CFU/ml)	5	3876667 (18300000)	0, 10000000	0	0	0
*Staphylococcus aureus* (CFU/ml)	2	170000 (912423.8)	0, 5000000	0	0	0
Coagulase-negative staphylococci (CFU/ml)	30	2473333 (3410120)	100000, 10000000	200000	1000000	3000000
*Candida albicans* (CFU/ml)	0	0	0	0	0	0

**Table 2:** Descriptive statistics for T1 phase

Variable	Positive counts	Mean (SD)	min, max	Q1	Median	Q3
Flow rate (ml/5 min)	–	7.93 (5.15)	2, 22.5	4	6.5	10
pH	–	7.67 (0.46)	6, 8.5	7.5	7.5	8
*Neisseria* spp. (CFU/ml)	30	1853333 (2347667)	100000, 10000000	300000	1000000	3000000
*S. mitis/oralis* (CFU/ml)	19	4400000 (9172109)	0, 50000000	0	2000000	6000000
*S. anginosus* (CFU/ml)	6	816666.7 (2219208)	0, 10000000	0	0	0
*S. mutans* (CFU/ml)	1	166666.7 (912870.9)	0, 5000000	0	0	0
*S. salivarius* (CFU/ml)	6	443333.3 (1306663)	0, 6000000	0	0	0
*S. sanguinis* (CFU/ml)	1	200000 (1095445)	0, 6000000	0	0	0
*S. parasanguinis* (CFU/ml)	5	733333.3 (2164499)	0, 10000000	0	0	0
*Staphylococcus aureus* (CFU/ml)	1	66666.67 (365148.4)	0, 2000000	0	0	0
Coagulase-negative staphylococci (CFU/ml)	30	1540000 (1743480)	100000, 8000000	300000	1000000	2000000
*Candida albicans* (CFU/ml)	0	0	0	0	0	0

**Table 3:** Descriptive statistics for T2 phase

Variable	Positive counts	Mean (SD)	min, max	Q1	Median	Q3
Flow rate (ml/5 min)	-	8.35 (4.83)	3, 24	5	7	11.5
pH	-	7.78 (0.28)	7, 8	7.5	8	8
*Neisseria* spp. (CFU/ml)	30	1456667 (1984541)	100,000, 7,000,000	300,000	400,000	2,000,000
*S. mitis/oralis* (CFU/ml)	19	3523333 (4305144)	0, 15,000,000	0	1,750,000	7,000,000
*S. anginosus* (CFU/ml)	6	836666.7 (2149656)	0, 8,000,000	0	0	0
*S. mutans* (CFU/ml)	1	166666.7 (912870.9)	0, 5,000,000	0	0	0
*S. salivarius* (CFU/ml)	6	690000 (2129165)	0, 10,000,000	0	0	0
*S. sanguinis* (CFU/ml)	1	166666.7 (912870.9)	0, 5,000,000	0	0	0
*S. parasanguinis* (CFU/ml)	5	1133333 (2800657)	0, 10,000,000	0	0	0
*Staphylococcus aureus* (CFU/ml)	2	46666.67 (185199.5)	0, 900,000	0	0	0
Coagulase-negative staphylococci (CFU/ml)	30	3850000 (4636865)	200,000, 20,000,000	500,000	2,000,000	7,000,000
*Candida albicans* (CFU/ml)	0	0	0	0	0	0

**Table 4:** Buffering capacity measurements

Time	Low	Medium	High	Total
T0	0	9	21	30
T1	1	7	22	30
T2	0	2	28	30

**Table 5:** Results of Friedman test

Variables	Statistic	p-value
Saliva buffering capacity	9.51	0.009
Saliva flow rate (ml/5 min)	9.80	0.006
pH	2.36	0.313
*Neisseria* spp. (CFU/ml)	1.87	0.389
*S. mitis/oralis* (CFU/ml)	0.57	0.780
Coagulase-negative staphylococci (CFU/ml)	2.92	0.213

**Table 6:** Results of the Wilcoxon signed-rank test

Variables	z-statistic	p-value
**Saliva buffering capacity**		
T0 – T1	0.000	1.000
T1 – T2	-2.646	0.0082
T0 – T2	-2.646	0.0082
**Saliva flow rate (ml/5 min)**		
T0 – T1	-1.789	0.0736
T1 – T2	-1.861	0.0628
T0 – T2	-3.313	0.0009

### Saliva Properties

**Fig 1:**
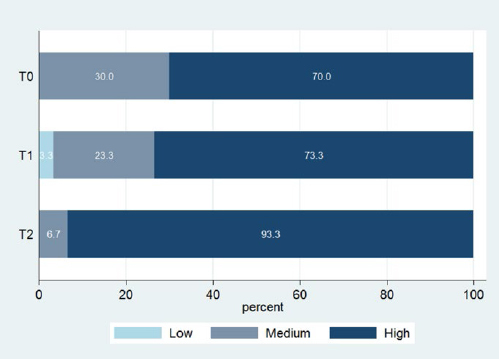
Saliva buffering capacity.

Saliva buffering capacity measurements by timepoint are displayed in Table 4 and the corresponding bar-plots are presented in Fig 1. Only one measurement yielded ‘low’ buffering capacity, which was measured at T1. Saliva buffering capacity seemed to increase at T2. Most measurements of the level ‘medium’ were identified at T0, whereas at T2, 28 of 30 outcomes were ‘high’. The Friedman test rejected the hypothesis that the distribution of buffering capacity across timepoints is the same (p=0.009). In detail, the Wilcoxon signed-rank test could not reject the hypothesis that the distribution of buffering capacity is the same between T0 and T1 (p≈1). However, there is evidence of an increase in buffering capacity between T1 and T2 as well as between T0 and T2 (p=0.008 for both comparisons).

**Fig 2:**
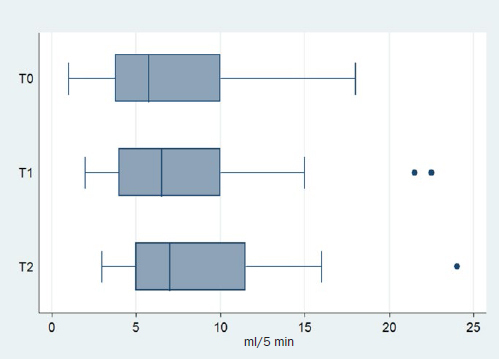
Box plots of saliva flow rate.

Descriptive statistics for flow rate at each timepoint are reported in Tables 1, 2 and 3. Mean flow rate appeared to increase with time. The Friedman test showed that the saliva flow rate changed with time (p=0.006). The Wilcoxon signed-rank test rejected the hypothesis that the flow rate between T0 and T2 was identical (p<0.001). On the other hand, it could not reject the hypothesis that the saliva flow rate is the same between T0-T1 and T1-T2. (p=0.07 and 0.06, respectively). Box-plots of the flow rate at each timepoint are shown in Fig 2.

**Fig 3:**
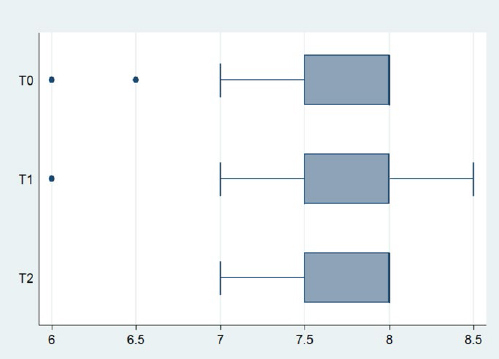
Box plots of salivary pH.

Salivary pH remained essentially unaltered throughout the study. A very small rise was detectable at T1 (7.67) and at T2 (7.78), but it was not statistically significant according to the Friedman test (p=0.313). The box-plots of the salivary pH are presented in Fig 3.

### Microbial Findings

Descriptive statistics for the various microbes identified at T0, T1, and T2 are reported Tables 1, 2, and 3 respectively.

**Fig 4:**
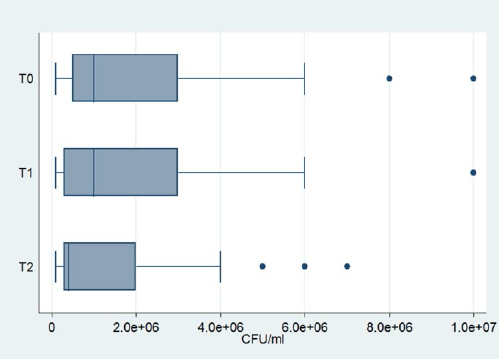
Box plots of *Neisseria* spp.

**Fig 5:**
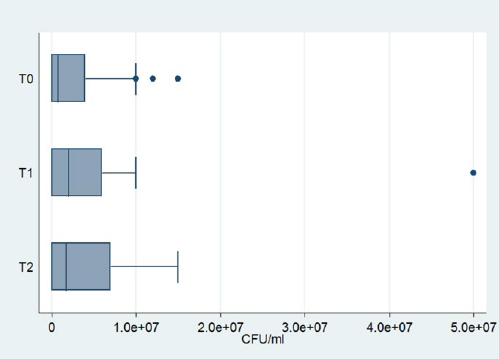
Box plots of *Streptococcus mitis/oralis.*

**Fig 6:**
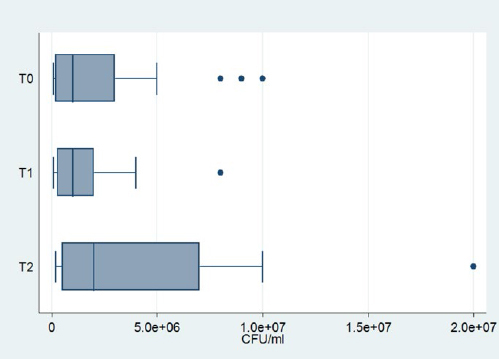
Box plots of coagulase- negative staphylococci.

- *Neisseria* spp. were identified in every measurement. The mean cfu decreased with time, but the Friedman test showed that this decrease was not statistically significant (p = 0.39). Box-plots of *Neisseria* spp. are given in Fig 4.
- *Streptococcus mitis/oralis* was identified in 19 patients at three times as shown in Tables 1, 2, and 3. At T1, an increase of the mean cfu was recorded, which then decreased at T2, almost reaching the levels of T0. This fluctuation was not statistically significant according to the Friedman test (p=0.78). Box-plots of *Streptococcus mitis/oralis* are shown in Fig 5.
- *Streptococcus anginosus* was identified in the same 6 patients at all three time-points. A slight decrease of the mean cfu at T1 was recorded and remained at these levels at T2.
- *Streptococcus mutans* was detected in only one patient at any time point. The initial level was up to 10^7^ cfu/ml followed by a reduction at T1 to 5x10^6^ cfu/ml. However, at T2, it remained steady.
- *Streptococcus salivarius* had 6 positive counts in the same patients at all timepoints. The mean cfu remained steady at T1, but a small increase was recorded at T2.
- *Streptococcus sanguinis* was detected in only one patient. A decrease was observed at T1, which continued at T2.
- *Streptococcus parasanguinis* was identified in the same 5 patients at all timepoints. There was a decrease of the mean cfu at Τ1, which, however, did not continue; on the contrary, there was an increase in Τ2, which did not however reach the initial levels.
- Coagulase-negative staphylococci were identified in all patients at all time points. A small decrease of mean cfu was recorded at T1, but this was followed by an increase at T2. Nevertheless, there was no statistically significant change according to the Friedman’s test (p = 0.213). Figure 6 shows the box-plots of CoNS.
- *Staphylococcus aureus* was found in only one patient at all three timepoints. A reduction was observed at T1, which continued at T2. It was also detected at T0 in one patient with a value of 10^5^ cfu/ml and at T2 in another patient with a value of 5x10^5^ cfu/ml.
- *Candida albicans* was not identified in any sample.

## Discussion

Buffering capacity is defined as the resistance to pH changes;^16^ this constitutes a physical salivary property which is responsible for the neutralisation of the acidic oral environment. Thus, it is able to control pH and slow the growth of microbes. Moreover, it reverses the low pH in plaque and it can act protectively against the demineralisation of the enamel.^16^ A high buffering capacity has been associated not only with low caries levels^11^ but also with caries resistance.^45^ Moreover, low buffering capacity has been correlated with a high caries prevalence^45^ and is considered to be a risk factor for the development of caries.^50^ In orthodontic patients with fixed appliances, most studies found no statistically significant difference in buffering capacity,^12,38,53^ while other studies reported either a statistically significant decrease^14,48^ or increase^15,34^ in the first period of orthodontic treatment.^12,38,53^ In the present study, the null hypotheses that saliva buffering capacity would not change from T0 to T2 was rejected (p=0.01). Buffering capacity appeared to increase after placement of fixed orthodontic appliances, especially between T1-T2 as well as T0-T2.

Saliva flow rate is also an important physical parameter that is essential for oral health, since it is considered to be a protective factor against caries.^17^ Moreover, a stimulated saliva flow rate <0.2 ml/min is considered to be abnormally low,^32^ and could be a potential risk factor for caries; low flow rate has been associated with caries development.^21^ Regarding orthodontic patients with fixed appliances, the results followed the same pattern as with buffering capacity; some studies found no statistically significant difference;^5,12,38^ whereas other showed an increase during treatment.^9,14,34,36,48^ According to our findings, the null hypothesis that saliva flow rate does not change from T0 to T2 was rejected (p=0.006). Saliva flow rate increased after the placement of fixed orthodontic appliances.

Low salivary pH has been associated not only with the presence of caries^45^ but also with periodontal disease.^8^ Our results indicated that there is no change in pH at the initial stages of fixed orthodontic treatment.

More than 500 species of microbes have been detected in the oral cavity.^47^ Many studies have shown the role of certain potentially pathogenic microbes on the occurence and evolution of caries^1,22,37,55^ which might also pose a threat to orthodontic patients.^6,20,23,33,40,46,49,57^ The current study found no statistically significant difference for any of the examined microbial flora.

Increased levels of different kinds of streptococci, such as *S. salivarius, S. sobrinus, S. parasanguinis* and *S. anginosus* are correlated with caries,^24,26,42^ while *S. mitis/oralis*, and *S. sanguinis* have a reverse relationship with the presence of caries. In the present research, *S. mutans* was predominantly absent since it appeared in only one patient. *Streptococcus salivarius* seemed to slightly increase at T2, while *S. parasanguinis* and *S. anginosus* levels appeared to decrease.

Concerning *Streptococcusi mitis/oralis*, *S. mutans, S. salivarius* and *S. sanguinis*, previous studies found an increase at 3 months of treatment with orthodontic appliances^54^ and a decrease after 6 months of orthodontic treatment after examining the subgingival plaque.^57^ In our study, *S. oralis* increased at T1, but decreased to the initial levels at T2, while *S. salivarius* increased at T2 and *S. sanguinis* decreased in both phases.

The findings on *Neisseria* and caries presence are contradictory: increased,^27^ decreased^26^ or unchanged levels^51^ have been found in different studies.

Staphylococcus is a genus of Gram-positive bacterium that colonises the oral cavity,^44^ appearing as various groups, such as coagulase-negative staphylococci (CoNS), with *S. epidermidis* and *S. haemolyticus* *being the most representative species*.^10^ The role of CoNS in oral diseases has not been fully explored; in the present study, CoNS was lower in all patients at T1 followed by an increase at T2, overcoming the initial levels. *S. epidermidis* is found to be prevalent in patients with caries.^18,28^

However, *Staphylococcus aureus* is excluded from this category and is also considered to be a potential pathogenic microbe associated not only with caries and gingivitis,^2,29^ but also with jaw osteomyelitis and parotitis.^13,31^ In the present research, *S. aureus* was found in only one patient at all timepoints, decreasing from baseline to T2.

## Conclusion

Buffering capacity appears to increase after the placement of fixed orthodontic appliances, especially between T1-T2 as well as T0-T2. Saliva flow rate also increased after the placement of fixed orthodontic appliances between T0-T2. This could have a protective action for orthodontic patients, in whom the appliance may influence oral hygiene. Moreover, our results indicate that there is neither a change in pH nor in the examined oral microbial flora in the initial stages of fixed orthodontic treatment. This prospective study, which controlled for confounding factors, may be regarded as preliminary research. A larger population and more advanced microbial detecting methods are needed for further detailed analysis. Thus, future studies to assess saliva properties and oral microbial flora in the longer term would be beneficial.
